# Best practice assessment methods for the undergraduate psychology program: a narrative review of the literature

**DOI:** 10.1080/00049530.2024.2395521

**Published:** 2024-09-01

**Authors:** Sarah Halliday, Peta Callaghan, Tiffany Lavis, Anna Chur-Hansen

**Affiliations:** School of Psychology, The University of Adelaide, Adelaide, Australia

**Keywords:** Undergraduate psychology, assessment methods, teaching, higher education, narrative review

## Abstract

**Objective:**

To determine if best practice guidelines exist for assessment methods throughout the undergraduate psychology program, and whether there are recommendations on how to scaffold these methods effectively and appropriately over the three-year degree.

**Methods:**

A comprehensive database search and review of journal articles, books, and grey literature relevant to higher education assessment and evaluation, and their focus on psychology, was conducted. From this, six articles, eight books, and one report were deemed relevant to the current research, with all but one published in the United States.

**Results:**

Four common themes arose from the findings: 1) assessments should be based on learning outcomes, 2) there are many considerations when creating assessments at a course and program level, 3) best practice assessment method: authentic assessment, and 4) recommendations for scaffolding assessment methods across the three-year program. Recommendations for scaffolding assessment methods related to the areas of scientific inquiry, psychological literacy, critical thinking, and problem-based learning.

**Conclusion:**

This review highlights that much more work is needed to identify the assessment methods that are best suited to each level in the undergraduate psychology program, and how to effectively scaffold and evaluate these methods over the three-year degree.

## Key points

**What is already known about this topic:**

1. Psychology educators in higher education often choose assessment methods for courses based on requirements and advice from accreditation boards and psychological associations, their personal pedagogical and teaching approaches, and through transgenerational influence, guided by more experienced educators’ opinions and approaches.
2. Common methods for assessing used inundergraduate psychology programs, that meet outcomes associated with the degree, include multiple choice question quizzes, essays or written assessment,oral presentations, portfolios, and authentic assessment.
3. Other disciplines, such as Medicine, provide educators with comprehensive handbooks based on scholarly research, which detail types of assessment methods and how to adapt these to various levels of study. Although there is some guidance available for psychology, this type of comprehensive resource does not exist for psychology educators.

**What this topic adds:**

1. The current review is the first to investigate and collate best practice recommendations for assessment methods in the undergraduate psychology program.
2. Our paper highlights important considerations related to assessment methods, as informed by the literature(such as the benefits of authentic assessment); however, in doing so, reveals that there are very limited widely accepted best practice recommendations that are consistently implemented in teaching practice and evaluated in research.
3. This paper provides a detailed call to action and recommendations for the undergraduate psychologyprogram, drawing on examples of comprehensive resources that exist in other fields.

Undergraduate psychology programs have become an increasingly popular choice for students wishing to pursue higher education (Clay, 2017; Steuer & Ham, 2008). In Australia, it is estimated that psychology is one of the top ten most popular study areas, comprising around 3.8% of the total graduate population (according to the 2023 Graduate Outcomes Survey) (Quality Indicators for Learning and Teaching [QILT], 2024). In the United States (US), estimates indicate that psychology is the fourth most popular major, with 1.2 to 1.6 million students taking introductory psychology classes each year (Clay, 2017). This popularity is echoed around the world, with estimates from Top Universities (2023) indicating undergraduate psychology is the 6^th^ most popular program globally. Given the substantial number of psychology students and graduates around the world, it is important to consider assessment methods in the psychology program to ensure uniform student experiences globally, and for employers to understand the capabilities and knowledge of psychology graduates.

In Australia, the undergraduate Bachelor of Psychology degree is typically a three-year program and is a pre-requisite for completing further education in the form of a fourth (or Honours) year, and for continuing onto specialised postgraduate studies (i.e., Masters or PhD). While the individual course structure and content of the undergraduate psychology programs vary between institutions around the world, most programs are guided by similar competencies, skills, and knowledge. Psychology boards across the United States (US), United Kingdom (UK), and Australia require psychology graduates to be able to describe the history and philosophy underpinning the science of psychology, explain key concepts and theories, and acquire the research skills and methods required to investigate psychological phenomena, as well as developing critical thinking skills, the ability to be culturally responsive, and demonstrate reflexive skills (American Psychological Association [APA], 2023; Australian Psychology Accreditation Council, 2019; The British Psychological Society, 2019). In some countries (in the UK and Australia, for example), these competencies, knowledge, and skills taught in undergraduate psychology programs are regularly monitored and assessed by accrediting boards or committees (APAC, 2019; The British Psychological Society, 2019).

Accredited psychology programs ensure that students receive a high-quality education where the learning outcomes, content, and assessments align with best practice guidelines and standards for registration as a psychologist (APAC, 2019; The British Psychological Society, 2019). In other countries where undergraduate psychology programs are not accredited, like in the US, the psychological association consistently updates and releases guidelines for educators (APA, 2023). Accreditation committees and psychological associations are similar in their approach of informing educators on contemporary, best practice guidelines for what should be covered in psychology programs, providing broad advice at a program, course, content, and assessment level. For example, The British Psychological Society (2019) provides the following direction for the design and implementation of assessments, “Programmes must have in place an assessment strategy that maps clearly on to programme and module learning outcomes, incorporates a wide range of formative and summative assessments, and which reflects students’ development of knowledge and skills as they progress through their studies” (p. 7). This advice is mirrored in an Australian setting, with APAC (2019) indicating, “the scope of assessment covers all program learning outcomes which include all graduate competencies for the relevant level(s)” and “there is a clear relationship between program learning outcomes and assessment strategies, which are criterion-based and ensure students demonstrate competence against all program learning outcomes” (p. 9). While it is understandable that this advice is given in a broad way, allowing for educators to adapt it to their own teaching practices and needs of their students, it does mean that assessment methods in undergraduate psychology programs vary across higher education institutions, both nationally and around the world.

Although there is no one definition of “assessment” in a higher education context, it is widely considered as the process of collecting information that helps measure the learning and achievement of students in a course and program (Carless, 2015; Gronlund, 2006). There are two main types of assessment, formative and summative. Summative assessments are used to measure student learning, by comparing student performance to a pre-determined standard or benchmark, whereas formative assessments are often used throughout the period of a course and offer students an opportunity to gain feedback from educators in order to learn, improve, and prepare for the summative assessments (Glazer, 2014). While both summative and formative assessments can apply to, and are important for, course and program learning outcomes, in many accredited undergraduate psychology programs it is important to ensure that students are meeting the benchmark of competency in a skill or knowledge base through the use of summative assessments (APAC, 2019).

In psychology programs, there are many types of summative assessments that educators can choose to implement in their courses (e.g., multiple choice questions, essays, research reports, case studies, reflections, etc.). In 2008, the American Psychological Association (APA) provided a comprehensive guide and critique of 40 assessment methods, considering the effectiveness when applied to common goals and outcomes of undergraduate psychology programs in the US (APA, 2008). The outcomes, as detailed by the APA (2008), are as follows: 1) knowledge base of psychology, 2) research methods in psychology, 3) critical thinking skills in psychology, 4) application of psychology, 5) values in psychology, 6) information and technological literacy, 7) communication skills, 8) sociocultural and international awareness, 9) personal development, and 10) career planning and development. As part of this report, the APA detailed common assessment methods used in a higher education institution and outlined the appropriateness of use for an undergraduate psychology program. While certain assessment methods met specific goals of the psychology degree more effectively (e.g., self-critiques and research teams were two of the more appropriate methods used to assess the goal of information and technological literacy; APA, 2008), other assessment methods met outcomes of the psychology degree more generally (e.g., essays or written pieces, oral presentations, portfolios, and authentic assessment). In addition to this APA report, there has been substantial work done in the space of creating and designing assessment methods to meet certain outcomes of the undergraduate psychology program. For example, Halonen et al. (2009) have provided detailed advice on how to teach critical thinking in psychology, and Einav et al. (2023) have recently detailed how applied scenarios can be used as a way to assess psychological literacy. This literature provides guidance regarding which assessments could be best suited to meet certain goals and outcomes of the psychology degree; however, it is not guaranteed that educators will choose these methods.

There are a range of possible explanations that can help discern why educators choose certain assessment methods to be delivered as part of their undergraduate psychology course. As highlighted, accreditation requirements guide the overall program learning outcomes that, in turn, guide the course learning outcomes at each level of study. As assessment methods should be based on the skills and knowledge associated with those learning outcomes (Rawlusyk, 2018), educators have some freedom in the types of assessment chosen to meet those outcomes. Although research has not yet examined how and why specific assessment methods are chosen by educators in higher education settings, it can be surmised that it may be based on educators’ own pedagogical practices and experience. Additionally, transgenerational influences may be at play, where assessment practices are informed by more experienced colleagues in the workplace. Although convenient and perhaps effective, the behaviours and knowledge of more experienced educators may not be informed by best practice or the literature. This is an issue for consistency across psychology programs, as well as a potential source for a lack of commonality in the skills and abilities of psychology graduates.

It also appears there is an absence of best practice guidelines that provide a uniform framework on how to scaffold assessment methods across three-year undergraduate psychology degrees, ensuring that students develop and build on their skills as they progress through the program. Best practice guidelines are not novel, with other disciplines (such as Medicine) providing comprehensive handbooks (see for example *A Practical Guide for Medical Teachers* (Dent et al., 2021)) to higher education teachers around the world, detailing the types of assessment methods that should be implemented and how to adapt these methods to suit students at varying levels in the undergraduate degree. Indeed, “Medical Education” is a specific field of scholarly enquiry, research, and practice, which covers undergraduate, postgraduate, and continuing professional development for students and practitioners of medicine (Swanwick, 2018). In comparison, whilst psychology scholars and teachers actively research approaches to pedagogy, evidenced by conferences such as AusPLaT (https://www.ausplat.com/), it can be argued that pedagogy research in this discipline is less developed and more recent than that from the field of medicine. Drawing on the practices of educators in medicine, psychology would benefit from having specific and widely used guidelines and handbooks that educators can draw on for assessing students in undergraduate programs, which are informed by contemporary evidence and best practice. This will contribute to ensuring that graduates have a consistent experience across institutions.

In sum, the popularity of psychology for undergraduate students necessitates uniform assessment methods to ensure consistent student experiences and clear understanding of graduates’ abilities for employers. Despite the broad guidelines provided by accrediting bodies and psychological associations, there is limited research on how and why specific assessment methods are chosen by educators in undergraduate psychology programs, suggesting variability in assessment methods across institutions. This raises concerns about consistency and quality of assessment practices, as this may lead to variability in the skills and competencies of psychology graduates, affecting the quality of education and employability. There are limited comprehensive best practice guidelines or resources for providing a uniform framework for scaffolding assessment methods through the undergraduate psychology degree.

Thus, the current research aims to review the literature to address two research questions:

1. What are the current best practices and guidelines for assessment methods in undergraduate psychology programs globally and within Australia?
2. What are the key gaps in existing literature regarding assessment practices in undergraduate psychology programs, and how can future research address these gaps to improve the quality and consistency of psychology education?

## Method

### Search strategy and inclusion criteria

To determine whether any guidelines exist outlining best practice assessment methods throughout the undergraduate psychology degree, the four members of the research team, assisted by an expert Liaison Librarian from the University of Adelaide, created a set of search terms for database searches in PsycINFO, ERIC, Scopus, and Google Scholar (see Appendix 1 for the full list of search terms). However, using terms like “psychology education” and “assessment methods” led to irrelevant results, such as articles on clinical psychological assessments, curriculum evaluations, student evaluations of teaching, or focused on other disciplines. After extensive and increasingly refined searches using Thesaurus terms, our final attempt to search the databases retrieved 121 results, which came from the same set of core journals as well as several books, book chapters, and grey literature. However, some of these were very dated, and the challenges associated with the database searches raised concerns about the systematic nature of our search. To address this, the team decided to focus the search within the set of identified core journals and hand-searched relevant articles’ reference lists to locate additional journals for review. Once these journals were identified, all reviewers used a consistent search strategy with the terms, “psychology”, “undergraduate”, “college”, and “assess*” (where “assess*” captures variations like “assessment(s)”, “assessed”, or “assessing”) to find articles specifically related to assessment methods in psychology degrees. Published work was eligible for inclusion if discussion was evident that provided best practice guidelines regarding assessment methods in the undergraduate psychology degree. Grey literature, in the form of published reports authored by recognised psychological associations, were also included if they addressed the research questions. All included pieces were required to be written in English.

**Figure 1.**
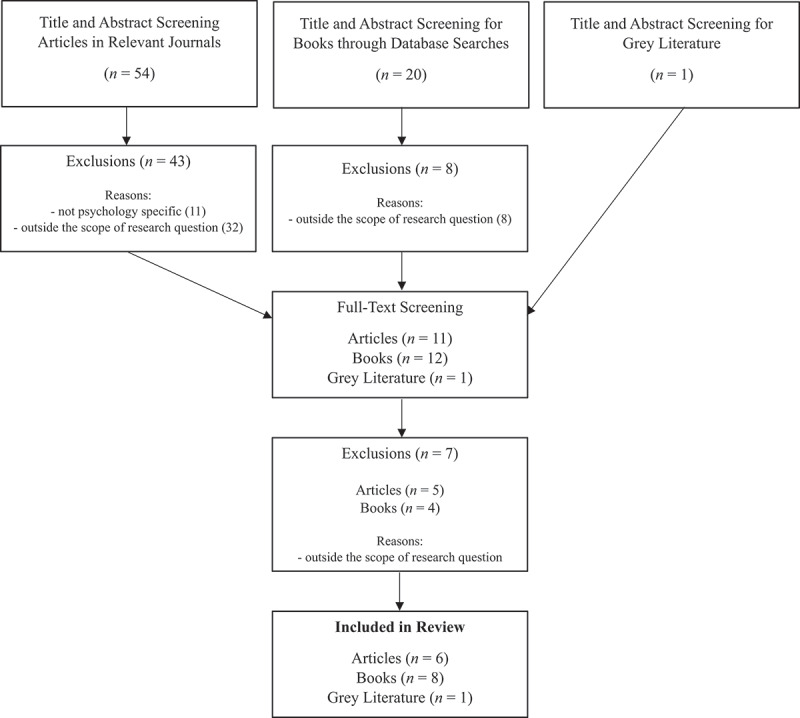
Flowchart detailing the findings from the literature search.

Twelve journals were chosen for review based on their relevance to higher education assessment and evaluation, and for their focus on psychology. These journals were: Psychology Learning and Teaching, Teaching of Psychology, Focus on Health Professional Education, Advances in Health Sciences Education, Psychology Teaching Review, Training and Education in Professional Psychology, Scholarship of Teaching and Learning in Psychology, Research in Higher Education, Higher Education, Assessment and Evaluation in Higher Education, Higher Education Research and Development, and Teaching in Higher Education. In addition to collecting relevant journal articles, the Liaison Librarian performed database searches for relevant books. The first author also searched for relevant grey literature, namely published and peer reviewed reports, from accredited psychological associations and leading societies in the field (such as the APS, BPS, and APA) (See Figure 1).

### Study selection and synthesis

Journals were divided among the research team for independent review which took place from 6 July 2023 to 31 July 2023. After screening titles and abstracts, relevant articles, books, and grey literature were collated in a shared document for the research team to confer on their eligibility for full-text review. Two reviewers independently conducted full-text screening, then consulted to determine inclusion. Title and abstract screening excluded *n =* 51 published works as *n* = 40 were outside the scope of the research question and *n* = 11 were not specific to psychology. Full-text article assessment excluded *n* = 7 for being outside the scope of the research question. While a formal quality appraisal was not conducted, the research team determined that the included books were from notable, respected sources and authored by scholars in the field, the journal articles had undergone a rigorous peer-review process, and the reports were sourced from esteemed, reputable associations in psychology education. A narrative approach was taken to synthesise and describe the collected data in a systematic and articulate way (Popay et al., 2006). This involved reporting on the aims and main findings relevant to the research questions, along with documentation of author(s), year and country of publication, and the title. Based on the descriptions of the main findings, the first author explored the overall ideas presented and subsequently grouped results by common concepts and themes. All four authors agreed on the chosen themes and presentation of data.

## Findings

A total of six journal articles, eight books, and one report met the criteria for inclusion in the present study. Table 1 details: the type of literature; the authors, year, and country of publication; the title; the aim of the book, article, or report; and summarises main findings from the article or key chapters relevant to the research questions. All but one of the included articles, books, and grey literature were US based. One book was published in the UK with three out of its four authors based in Cyprus (Papageorgiou et al., 2015). There was no identified literature from Australia. Common themes that arose from the literature regarding best practice guidelines in undergraduate psychology programs are as follows: 1) assessments should be based on learning outcomes, 2) there are many considerations when creating assessments at a course and program level, 3) best practice assessment method: authentic assessment, and 4) recommendations for scaffolding assessment methods across the three-year program.

**Table 1.** Summarised findings from the literature review.

Type of Literature	Author (Year) Country	Title	Aim	Main Findings
Book	Dunn et al. (2004) United States	Measuring up: Educational assessment challenges and practices for psychology	To provide concrete practices for student assessment in psychology education, measuring achievement, and promoting quality instruction in psychology.	• Chapter 1: Highlighted how learning outcomes should be based on student learning and developed over the program. Provided broad outcomes that can be assessed in psychology programs. These outcomes included content knowledge of the discipline, methods and basic research concepts, language and literacy skills, critical thinking skills, information gathering and synthesis, knowledge of and appreciation for the arts, skills gained through practical experience, ethics and values, interpersonal skills. • Chapter 6: This chapter drew on the theory of successful intelligence to explain how some students achieve more when assessments are varied (for example, using memory based, analytical, or practical assessments). • Chapter 8: This chapter described how authentic assessment can be used to assess scientific inquiry at the basic, developing, integrating, and professional level. Provided a developmental rubric that could be used to guide students to a mastery level. • Chapter 9: Similar to the previous chapter, this chapter focused on various levels of ability/proficiency but in relation to self-assessment.
	Dunn et al. (2008) United States	Teaching critical thinking in psychology: A handbook of best practices	To provide best practices for teaching critical thinking issues in psychology courses.	• Chapter 6: Outlined critical thinking requirements in assessment and provided tools for assessing the construct. Both assessments that were provided are MCQ based. • Chapter 7: Detailed a case study of James Madison University’s approach to assessing critical thinking at a program level. Outlined the use of behavioural checklists and student reflections as part of this process.
	Dunn et al. (2013) United States	Assessing teaching and learning in psychology: Current and future perspectives	This book is designed to be a helpful response to the assessment needs of psychology teachers, department heads, and program administrators in 2-year and 4-year institutions.	• Chapter 2: This chapter drew on the Educational Assessment Model to answer questions relating to the pressures that shape the delivery and assessment of undergraduate psychology education and the current perceptions about the preparation of college students in relation to the learning outcomes. Discussions focused on the importance of considering how graduates should be effective in the workplace, being responsible citizens, and possessing critical writing and thinking skills. Assessments were discussed in the context of ensuring alignment to course learning outcomes. • Chapter 3: This chapter highlighted the importance of creating meaningful rubrics for assessment and then provided a top-level description of embedded assessment, authentic assessment, and rubrics, before describing specific assessments that had been implemented in courses. There were no suggestions related to how to appropriately scaffold assessments across the program in this chapter. • Chapter 9: This chapter outlined how to assess psychological literacy in undergraduates. The authors briefly touched on the importance of authentic assessment and how this should drive the curriculum, in the sense that the tasks that students need to perform should be progressively developed through the curriculum (“planning backwards”). Specifically looking at psychological literacy, the authors first defined the concept using the work of McGovern et al. (2010), before suggesting how to show evidence of students obtaining this skill. They proposed the idea of introducing a graduate attribute portfolio that is introduced in first year based and is used in each psychology course for the remainder of the program.
	Gurung and Neufeld (2022) United States	Transforming introductory psychology: Expert advice on teacher training, course design, and student success	This book is a compilation of recommendations developed by members of the APA Introductory Psychology Initiative surrounding the areas of student learning outcomes and assessments, course models and design, teacher training and development, and student success and transformation.	• Although this book predominately focused on introductory psychology, there were suggestions related to assessment design. • Chapter 2: Described backwards design when considering learning outcomes and assessments. • Chapter 3: Gave recommendations for the proficiency of students in the introductory psychology level in various areas: biopsychology, cognitive, development and learning, social and personality, mental and physical health, and how assessments can then build on these concepts. • Chapter 4: Provided examples of types of assessments that could be implemented, with these based on the APA guidelines (version 2.0); however, this did not include information related to benchmarking, scaffolding across levels, offer evidence of effectiveness, or specify desirable outcomes. Also provided broad level advice for questions to consider when designing assessments.
	Mayo (2010) United States	Constructing undergraduate psychology curricula: Promoting authentic learning and assessment in the teaching of psychology	This instructional resource presents a framework for constructing undergraduate psychology curricula to meet all of the following objectives while ensuring maximum flexibility across Faculty, student, and institutional differences. The objectives are knowledge base of psychology, research methods, critical thinking skills, application of psychology, and values of psychology.	• Chapter 3: Introduced authentic assessment and explained how these can replace the rote learning/passive test taking, which occurs in multiple choice, matching, or true-false questions. • Chapter 4: Detailed how case-based instruction can be used in undergraduate psychology. Provided assessment examples of various case-studies which included details on the instruction, the proficiency level it is targeted to, associated learning outcomes, and a guide for rubric creation.
	Neff and Donaldson (2012) United States	Teaching psychology online: Tips and strategies for success	This book shows readers how to effectively create and manage an online psychology course. Guidelines for preparing courses, facilitating communication, and assigning grades are provided along with activities and assessments specifically geared towards psychology.	• Chapter 6: This chapter detailed best practices for the transition to online assessment by considering learning outcomes, summative versus formative assessment, and learner centred approach to assessment. There were brief discussions related to types of assessment that could be implemented in online courses: reflections and self-assessment, case studies, class opinion polls, self-confidence surveys. The authors suggested that online assessments should be authentic, challenging, coherent, engaging, respectful, responsive, rigorous, and valid. There were suggestions of assessment that are not effective online (pop-quizzes). Tips were provided for implementing assessments online and factors to consider in online environments.
	Nolan et al. (2021) United States	Assessing undergraduate learning in psychology: Strategies for measuring and improving student performance	This book shows how to develop assessments that undergraduate psychology faculty and administrators can use when designing pedagogies, courses, and curricula around student learning goals.	• Chapter 4: Briefly described how members of Faculty can share assessment strategies across a program to promote a stronger sense of ownership and should embed assessment in existing coursework. • Chapter 9: Provided a useful table for mapping assessments onto learning goals of a course or program.
	Papageorgiou et al. (2015) United Kingdom/Cyprus	Psychology for psychologists: A problem-based approach to undergraduate psychology teaching	This book uses psychological theories and learning processes, such as Problem Based Learning (PBL), to provide a new approach for teaching psychology at an undergraduate level and prevent diminishing motivation. It creates a detailed example of a psychology degree using the PBL method and suggests how a week of the course could be planned.	• Chapter 2: This chapter provided step-by-step instructions on how to implement PBL in your classroom and the types of topics that could be covered. • Chapter 3: This chapter specifically focused on assessment design with the authors outlining the basic principles behind a fair, successful, and reliable assessment using PBL, and provided examples for various types of assessment. • Chapter 4: Detailed how PBL can be drawn on for each year in the undergraduate psychology degree (curriculum and assessment). This chapter showed how scaffolding of PBL can occur in each year level.
Journal Article	Altman et al. (2021) United States	Reimagining how we teach introductory psychology: Support for instructors adopting the recommendations of the APA Introductory Psychology Initiative	This article aims to provide models that will enable both novice and experienced instructors to integrate IPI’s student learning outcomes and offers examples of assignments that instructors can adopt immediately.	• The authors highlighted the importance of backwards design and course learning outcomes in the creation of assessments. • Detailed the process of backwards design then provided specific examples of summative assessments that may be useful in the introductory psychology course. This included semester-long projects, scaffolded hands-on research projects, and “big problems” which can use discussion boards as its platform. • Provided potential student learning outcomes for the introductory course.
	Burns et al. (2020) United States	Culturally responsive assessment in the psychology college classroom	This article provides an overview of culturally responsive pedagogy and assessment and describes how these practices can be translated to the college classroom.	• This paper was guided by the APA Guidelines (version 2.0) and detailed how assessments focused on being culturally responsive can be created by involving the students, reconceptualising assessment and allowing various modalities, examining equity in the classroom, and increasing transparency and accessibility. • There were no suggestions regarding how to scaffold assessments focusing on this psychological skill/knowledge across the year levels.
	Dunn et al. (2020) United States	Doing assessment well: Advances for undergraduate psychology programs and psychology educators	This article discusses how assessment tools can be used to improve student learning in undergraduate bachelors (BA/BS) in psychology programs.	• Provided resources for assessment development from the APA. • Described course sequencing in the context of assessments and detailed how some courses should be pre-requisites for others. For example, courses offered earlier in the psychology major should provide more basic information and skills, while those in the middle and end of the curriculum should be more challenging, containing advanced material that assumes student understanding of earlier subject matter, as well as the acquisition of particular skills (e.g., research design skills in methods and interpretative skills in statistics/data analysis). • Provided broad detail regarding the following assessment methods: major projects, oral presentations, final (exit) exams, group projects, portfolios.
	Halonen et al. (2003) United States	A rubric for learning, teaching, and assessing scientific inquiry in psychology	This article explores how to meet the pressures of accountability with integrity, focusing on authentic assessment and teaching as a primary solution. The authors propose a rubric to describe the progress of students’ acquisition of scientific inquiry skills.	• The authors proposed a developmental rubric based on the following domains of scientific inquiry in the field of psychology: descriptive skills, conceptualisation skills, problem solving skills, ethical reasoning, scientific values and attitudes, communication skills, collaboration skills, and self-assessment. • For each domain, authors described the expected abilities of students in varying proficiency levels including before training, basic introductory psychology, developing, integrating advanced undergraduate, professional graduate and beyond. • Authentic assessment was recommended for assessment design.
	Ishak and Salter (2017) United States	Undergraduate psychological writing: A best practices guide and national survey	This article provides a best practice guide for teaching psychological writing beyond APA style, discusses psychology-specific writing assignments, and examines psychological writing instruction.	• Although the focus was largely on teaching, there were recommendations on how to assess psychological writing. • To scaffold, authors suggested focusing assessments onto Bloom’s Taxonomy and to use authentic assessment and critical thinking assignments. • Other considerations and advice were mostly at a top level.
	Rimzhim et al. (2020) United States	One psychology department’s home-grown programmatic assessment of their curriculum	This article details the design, implementation, and results of a five-step curricular assessment initiative.	• The authors of this paper provided a holistic view on assessment creation at a program level. This model was described as “evidence-based, multistep, comprehensive, in-house, and sustainable”. • Faculty were required to map individual courses’ curriculum and assessment onto agreed upon department learning outcomes. • The model was used to improve student learning. • The authors and Faculty reported this was a time consuming and labour-intensive approach to gathering program level information.
Grey Literature	American Psychological Association (2023) United States	APA guidelines for the undergraduate psychology major: Empowering people to make a difference in their lives and communities	This report aims to provide a comprehensive revision of the national recommendations regarding the knowledge and skills that undergraduate psychology majors should acquire at the associate and baccalaureate levels of study in undergraduate psychological science programs.	• This report included guidelines and recommendations regarding the knowledge and skills undergraduate psychology majors should acquire in the US. • There were “foundation indicators” and “baccalaureate indicators” that indicated what students should be able to demonstrate at those levels. • Broad advice was given regarding assessment creation which included collaborating with everyone involved in delivering the program when decision-making, considering where psychology students end up in their careers (authentic assessment), how to incorporate science into research courses, and the importance of diversity, equity, and inclusion.

### Assessments should be based on learning outcomes

One of the most frequent points raised was the importance of creating assessments that are derived from, and align with, the course or program learning outcomes (Altman et al., 2021; Dunn et al., 2004, 2013; Gurung & Neufeld, 2022; Ishak & Salter, 2017; Neff & Donaldson, 2012; Nolan et al., 2021; Rimzhim et al., 2020). This finding is not new, rather it is quite prevalent among higher education literature, especially when discussing the design process and implementation of assessments (Carless, 2015; Gronlund, 2006). Although this finding is somewhat unsurprising, it is reassuring that one of the most common best practice guidelines that exists for creating assessments in higher education (i.e., assessments are informed by learning outcomes) is also heavily referenced in psychological literature on teaching and learning assessment considerations.

A common framework that is based on the importance of considering course learning outcomes in higher education is the backward design model, which was also discussed in the included literature (Altman et al., 2021; Dunn et al., 2013; Gurung & Neufeld, 2022). Backward design refers to focusing first on the learning objectives or outcomes, and encourages intentionality in the planning and delivery of the course, including assessments (Wiggins & McTighe, 1998). In Chapter 9 of Dunn et al. (2013) book, the authors describe how backward design (referred to as “planning backwards”) should be progressively developed through the curriculum, and they provide an example of portfolio assessments that can be scaffolded throughout the three-year degree. While the included pieces detailed what backward design is, and its usefulness in the context of higher education assessment creation, there remains a gap in the literature that guides educators on how to develop learning outcomes throughout the three-year psychology degree, based on the expected skills and knowledge of students, which then informs assessment creation at each level. This highlights the expectation that teachers in higher education settings, especially in psychology, create their own learning outcomes based on the requirements of the individual institution, rather than being informed by evidence.

### Other considerations when creating assessments at a course and program level

With much of the literature published in the US, there is a prominent emphasis on using the APA guidelines for the undergraduate psychology major to inform assessment design and implementation (APA, 2023). The most recent guidelines, version 3.0, had only just been released at the time of writing (August 2023), so previous versions from 2013 (version 2.0) and 2006 (version 1.0) were used and referred to in the books and papers that form this review (Burns et al., 2020; Dunn et al., 2020; Gurung & Neufeld, 2022). Specifically focusing on the assessment guidelines in the current version, the APA provides contemporary, yet broad advice for designing assessments, including the importance of considering psychology graduates’ destinations (and how wide-ranging career options are), how to incorporate open science into research courses, and the importance of internationalisation, equity, diversity, and inclusion (APA, 2023). Literature drawing on advice of previous versions of the APA guidelines also suggest best practice for assessments should consider being culturally responsive, co-creation with students, allowing for various modalities, increasing transparency and accessibility (Burns et al., 2020), and involving course-sequencing where assessments earlier in the program are pre-requisites for later, more challenging assessments, drawing on more advanced knowledge and skills (Dunn et al., 2020). The APA guidelines and recommendations provided by experts in the area are valued and important in the discussion of higher education assessment design; however, the broad context in which these suggestions are presented allow for discrepancy across institutions, and even between educators in the same institution. It is also important to acknowledge that there has been no research that draws on the guidelines of accrediting boards (like APAC in Australia) to create best practice guidelines and suggestions of how to scaffold assessment methods across the psychology degree as a resource for educators.

Another important consideration for creating assessments at a course and program level is how to translate face-to-face assessments to an online format; however, only one book examined this topic in detail (Neff & Donaldson, 2012). The authors suggest that best practice for transition to online assessments includes considering learning outcomes, when summative or formative assessments are most appropriate, and having a learner centred approach. Like face-to-face assessments, online assessments should still be authentic, challenging, coherent, engaging, respectful, responsive, rigorous, and valid (Neff & Donaldson, 2012). The authors also suggest that reflections and self-assessment, case studies, class opinion polls, and self-confidence surveys would be appropriate in online contexts, while assessments such as pop-quizzes are not appropriate. General tips for teaching online are provided, but given the ever-changing landscape of technology, some are outdated, and current issues concerning assessments in higher education are not discussed (such as the impact of artificial intelligence).

### Best practice assessment method: authentic assessment

From the included literature, a widespread best practice recommendation for assessing in undergraduate psychology programs is incorporating authentic assessment (APA, 2023; Dunn et al., 2004, 2013, 2020; Halonen et al., 2003; Ishak & Salter, 2017; Mayo, 2010; Neff & Donaldson, 2012). Authentic assessment aims to promote abilities related to employability and future careers by replicating tasks in real-life experiences and performance standards typically found in a work environment (Villarroel et al., 2018). These types of assessments are shown to improve student learning, autonomy, motivation, self-regulation, and metacognition (Villarroel et al., 2018).

While there is no specific advice in the included literature that explains how to effectively scaffold authentic assessment across the three-year program, or how to design a reliable authentic assessment for varying skill and knowledge levels of students, there are some important factors related to authentic assessment that are pertinent to psychology educators. In Chapter 4 of *Constructing Undergraduate Psychology Curricula Promoting Authentic Learning and Assessment in the Teaching of Psychology* (Mayo, 2010), the author explains how case studies in psychology programs can be used as forms of authentic assessment, aligning with the learning outcome of *demonstrate critical thinking skills in psychology*. An example that is provided details a scenario and asks students to comment on, and relate the scenario to, theoretical psychological applications (i.e., biological, cognitive, cross-cultural, and behavioural perspectives). This encourages students to make connections between theoretical and applied knowledge and is shown to be an effective teaching strategy in psychology courses (Thistlethwaite et al., 2012).

While it is important for psychology programs to create authentic assessments based on psychological perspectives and theories, recent suggestions extend the trend of primarily focusing on clinical psychology-based scenarios. The APA (2023) highlights that many graduates from baccalaureate psychology programs in the US do not go on to pursue masters or doctorate degrees in the field (i.e., less than 14% of psychology graduates). Similarly, in Australia the majority of undergraduate psychology students will not continue after the Bachelor degree, and those who do may pursue a range of different degrees and careers (see for example, https://psychology.org.au/careers). Therefore, authentic assessment should also draw on the broad range of skills, knowledge, and competencies that are valued in the workforce. These include critical thinking and problem-solving skills; locating, evaluating, and using information decision-making; analysing and interpreting data; making sound decisions and ethical judgement; and communicating and working with people from different backgrounds using various formats, like writing and presentations (APA, 2023). Given the effectiveness of authentic assessment in psychology programs, it would be beneficial to incorporate more work-ready skills and knowledge alongside psychological theories and perspectives, and should be considered in future practice.

### Current recommendations for scaffolding assessment methods across the program

The second research question focuses explicitly on whether there are best practice guidelines that detail how to scaffold assessment methods across the three-year psychology program. Drawing on the teaching practices of other fields (such as Medicine), the psychology discipline would benefit from clear and evidence-based instruction on how to scaffold assessment methods over the three-year degree, specifically detailing the methods that are most appropriate for the skills, knowledge, and abilities of each year level. This need is somewhat (but not completely) addressed in the included literature (Dunn et al., 2004, 2008, 2013; Halonen et al., 2003; Mayo, 2010; Papageorgiou et al., 2015).

This review found scaffolding across the three-year program advice present for assessing broad areas related to studying a degree in psychology, including scientific inquiry (Dunn et al., 2004; Halonen et al., 2003), critical thinking (Dunn et al., 2008), psychological literacy (Dunn et al., 2013), and problem-based learning (Papageorgiou et al., 2015). Some authors provided recommendations for scaffolding by explaining proficiency levels that related back to learning outcomes (Dunn et al., 2004, 2013; Halonen et al., 2003), while others chose to provide example behavioural checklists for different levels (Dunn et al., 2008). One example of proficiency level descriptions was provided by Dunn et al. (2004) who detailed scientific inquiry at varying proficiency levels (i.e., basic, developing, integrating, and professional) for factors in the domains of descriptive skills, conceptualisation skills, problem solving skills, ethical reasoning, scientific attitudes and values, communication skills, collaboration skills, and self-assessment skills. While these descriptions are detailed, there are no suggestions on the best assessment methods to use for each domain at each proficiency level.

Papageorgiou et al. (2015) address this limitation, and, in their focus on problem-based learning in psychology, provide explicit instruction on how to embed problem-based learning into curriculum and assessment at each year level. First, psychological themes for each year are proposed along with their learning outcomes, then options for assessment are provided with an itinerary of when to implement them throughout the semester or year and how it builds on the previous year. One drawback is that the authors suggest placements should be implemented from, and build on, the first year as a form of assessment. This is an issue in Australia given placements are not routinely included or formally required in undergraduate psychology programs. Additionally, securing placement locations can be challenging, with priority given to those completing postgraduate degrees (such as a master’s degree) (Psychology Board of Australia, 2022). When placements are included in undergraduate degrees, they require diverse locations and skillsets, with students having a broad range of abilities and interests, and therefore, assessment methods should be considered in light of these factors and contexts.

One study that focused on scaffolding assessment methods detailed how portfolios can be used to build on learning from each year of the undergraduate psychology program (Dunn et al., 2013). The authors suggest that a graduate attribute portfolio be used and structured in a way that the student is required to reflect on what they already know, what they are learning in that unit, how the content impacts their life outside the unit, what else they need to accomplish, and their plans to achieve that end. They also suggest that each psychology unit throughout undergraduate years have a small assessable activity that leads to the further development of this portfolio with increased attention towards the end of the undergraduate program.

### Limitations

This study aimed to review the current literature on best practices for assessment methods in undergraduate psychology programs. Due to the inclusion criteria and search strategy employed, many of the sources and findings were theoretical in nature. For example, concepts such as alignment of assessments to course learning outcomes, and the backward design process. As a result of the scarcity of empirical evidence that addressed the aims of this review, the conclusions drawn from the findings relied heavily on theoretical frameworks. Further empirical research is required to identify the most effective assessment methods, and how these can be scaffolded, to improve the experiences and outcomes for undergraduate psychology students. Nearly all of the included sources in this review were published in the United States, with a bias towards the US undergraduate psychology program. While the content across US psychology programs shares similarities to those in Australia (APA, 2023; APAC, 2019), there are considerable differences in program expectations. In Australia, for instance, educators must adhere to specific accreditation standards set by APAC, whereas accreditation is not mandatory for US programs. Consequently, while the insights gained from this review can offer valuable perspectives, it is important to recognise that assessment methods may vary across countries due to differing accreditation requirements.

Overall, this review identified valuable and informative recommendations on best practice guidelines related to assessment practices in undergraduate psychology programs. Specifically, the importance of ensuring alignment between assessments and learning outcomes of the institution and accrediting boards or psychological associations, effective use of authentic assessment, and guidelines for scaffolding across the degree related to some psychological theories and perspectives. While this study provided broad, yet helpful, information regarding the current state of assessment practices in the undergraduate psychology degree, the limited work providing explicit and evidence-based recommendations is a concern.

## A call to action

As noted, other disciplines have well established approaches to the development and evaluation of teaching and learning methodologies, including assessment practices. The Australian Medical Council (AMC), for example, is explicit regarding types of assessments to be used in university curricula (see, for example, https://www.amc.org.au/wp-content/uploads/accreditation_recognition/specialist_edu_and_training/assessment/standards_for_assessment.pdf). The Australian Psychology Accreditation Council (APAC) is less prescriptive regarding assessment practices, which although allows for creative freedom as an educator, does introduce a risk of inconsistency and discrepancy for psychology programs in Australia. Psychology as a discipline has, as a core foundation, the notion of evidence to inform practice. It is curious that over the decades, psychology teachers in higher education settings seem to have contributed less, than one might imagine, to researching their teaching practices and gathering evidence regarding student outcomes. For example, we are not aware of longitudinal studies that follow students from the entry of the degree to graduation and employment, to determine the predictive validity of assessments. There are logistical challenges to such research, as well as ethical challenges in researching one’s students (see Thomas et al. (2019) for an outline); however, given the need for evidence to inform best practice teaching, and the need to disseminate best practice through research, we argue that barriers and challenges should not be reasons to accept the status quo.

One major limitation that still exists is the lack of psychological research that details the assessment methods that are best suited to each year level of the degree. For example, whether it is most appropriate to use multiple choice questions (MCQs) in first year to build basic student knowledge and skills for larger cohorts, before extending these to more advanced methods of assessment in the second and third years (such as applied short-answer questions, research reports, and case-based scenarios). Furthermore, in light of artificial intelligence, are MCQs, and particularly recall of information approaches (as well as other assessments) still viable? Future efforts in this field should work to build up the literature base regarding best practice recommendations for assessment methods in undergraduate psychology programs, before working towards building a practical, evidence-based handbook. This proposed handbook should also align with the competencies and requirements of accrediting boards, like APAC in Australia, to ensure consistency in teaching practices. Providing explicit direction would ensure educators are teaching similar skills to psychology graduates not only in the same institution, but also nationally, and eventually, internationally.

### Recommendations

Based on our review, we make the following recommendations:

#### Development of a robust evidence base

A concerted effort should be made to continue to develop a robust evidence base for assessment methods in the undergraduate psychology program, aligned with relevant accreditation requirements. While existing literature emphasises the alignment of assessments with learning outcomes, it lacks detailed guidance on developing these outcomes and assessment methods in an evidence-based way. This gap underscores the need for more comprehensive research, including, where possible, longitudinal studies to follow psychology students from entry into the degree through to graduation and employment.

#### Creation of a best practice psychology assessment framework

Efforts should be made to develop a best practice “psychology assessment framework”, which considers important factors such as scaffolding throughout the degree, the proficiency and skill level of students in each year, appropriate and authentic assessment methods, and accreditation requirements. Although there are broad guidelines from accrediting bodies, they do not provide specific, actionable strategies, thereby justifying the need for a detailed, practical framework.

#### Detailed scaffolding strategies

Consideration should be given to detailed scaffolding from level 1 to level 3 of the undergraduate program, taking into account course and program learning outcomes, authentic assessment, diversity and inclusion, and other factors not included in this review, such as psychological literacy, rubrics, and marking. Current literature mentions scaffolding but lacks specific strategies for its implementation across different year levels, therefore discussions about scaffolding need more precise and evidence-based guidance.

#### Addressing online teaching and AI impact

Attention should be paid to the benefits and challenges of online teaching and the impact of artificial intelligence on assessment design. Existing research on online assessment practices needs considerable attention now regarding the implications of AI. There is a strong need for current and comprehensive guidelines in this area.

## Data Availability

The authors confirm that the data supporting the findings of this study are available within the article and its supplementary materials.

## Appendix 1

Terms used in preliminary database searching

### PsycINFO and Scopus

**Table:**

Psychology Education	Assessment methods
Exp Psychology education OR Introductory Psychology Education.sh OR undergraduate psychology education.sh OR (psychology education OR psychology program* OR psychology course* OR psychology student* OR psychology undergraduate* OR undergraduate psychology).ti,ab,id	Educational measurement.sh OR Curriculum based assessment.sh OR formative assessment.sh OR “grading (educational)”.sh OR minimum competency tests.sh OR (educational measurement* OR educational testing OR curriculum based assessment* OR formative assessment* OR summative assessment* OR performance assessment* OR classroom assessment* OR formative performance assessment* OR summative performance assessment* OR peer assessment* OR educational grading OR competency test* OR skills test* OR written assignment* OR written report* OR rubric* OR “evidence of student learning” OR student assessment* OR quiz* OR exams OR examinations OR essay* OR “student* knowledge of content” OR embedded question* OR embedded assignment* OR oral presentation* OR standardi*ed test* OR self-critique* OR group project* OR multiple choice question*).ti,ab,id

### ERIC

TI,AB(“psychology education” OR “psychology program*” OR “psychology course*” OR “psychology student*” OR “psychology undergraduate*” OR “undergraduate psychology”) AND MJMAINSUBJECT.EXACT.EXPLODE(“Assignments”) OR MJMAINSUBJECT.EXACT(“Student Evaluation”) OR MAINSUBJECT.EXACT(“Writing Evaluation”) OR MAINSUBJECT.EXACT(“Portfolio Assessment”) OR MAINSUBJECT.EXACT(“Educational Testing”) OR MAINSUBJECT.EXACT(“Informal Assessment”) OR MAINSUBJECT.EXACT(“Performance Based Assessment”) OR MAINSUBJECT.EXACT(“Grading”) OR MAINSUBJECT.EXACT(“Grades (Scholastic)”) OR MAINSUBJECT.EXACT(“Peer Evaluation”) OR MAINSUBJECT.EXACT(“Scoring Rubrics”) OR MAINSUBJECT.EXACT(“Holistic Evaluation”) OR MAINSUBJECT.EXACT(“International Assessment”) OR MAINSUBJECT.EXACT(“Alternative Assessment”) OR MJMAINSUBJECT.EXACT(“Formative Evaluation”)
